# Multiscale habitat relationships of snowshoe hares (*Lepus americanus*) in the mixed conifer landscape of the Northern Rockies, USA: Cross‐scale effects of horizontal cover with implications for forest management

**DOI:** 10.1002/ece3.2651

**Published:** 2016-12-14

**Authors:** Joseph D. Holbrook, John R. Squires, Lucretia E. Olson, Rick L. Lawrence, Shannon L. Savage

**Affiliations:** ^1^ USDA Forest Service, Rocky Mountain Research StationMissoulaMTUSA; ^2^ Department of Land Resources and Environmental SciencesMontana State UniversityBozemanMTUSA

**Keywords:** gradient modeling, habitat selection, habitat use, occupancy, quantile regression, Random Forest

## Abstract

Snowshoe hares (*Lepus americanus*) are an ecologically important herbivore because they modify vegetation through browsing and serve as a prey resource for multiple predators. We implemented a multiscale approach to characterize habitat relationships for snowshoe hares across the mixed conifer landscape of the northern Rocky Mountains, USA. Our objectives were to (1) assess the relationship between horizontal cover and snowshoe hares, (2) estimate how forest metrics vary across the gradient of snowshoe hare use and horizontal cover, and (3) model and map snowshoe hare occupancy and intensity of use. Results indicated that both occupancy and intensity of use by snowshoe hares increased with horizontal cover and that the effect became stronger as intensity of use increased. This underscores the importance of dense horizontal cover to achieve high use, and likely density, of snowshoe hares. Forest structure in areas with high snowshoe hare use and horizontal cover was characterized as multistoried with dense canopy cover and medium‐sized trees (e.g., 12.7–24.4 cm). The abundance of lodgepole pine (*Pinus contorta*) was associated with snowshoe hare use within a mixed conifer context, and the only species to increase in abundance with horizontal cover was Engelmann spruce (*Picea engelmannii*) and subalpine fir (*Abies lasiocarpa*). Our landscape‐level modeling produced similar patterns in that we observed a positive effect of lodgepole pine and horizontal cover on both occupancy and use by snowshoe hares, but we also observed a positive yet parabolic effect of snow depth on snowshoe hare occupancy. This work is among the first to characterize the multiscale habitat relationships of snowshoe hares across a mixed conifer landscape as well as to map their occupancy and intensity of use. Moreover, our results provide stand‐ and landscape‐level insights that directly relate to management agencies, which aids in conservation efforts of snowshoe hares and their associated predators.

## 1 Introduction

The conservation of strong interactions within food webs is important to sustain ecological stability and biological diversity (e.g., McCann, Hastings, & Huxel, 1998). Snowshoe hares (*Lepus americanus*) interact strongly with multiple species in the boreal forests of North America. For instance, snowshoe hares represent 48% of the vertebrate biomass and 41% of the mean energy flow in the southwestern Yukon (Krebs, Boutin, & Boonstra, 2001). Predators such as coyotes (*Canis latrans*), American marten (*Martes americana*), red fox (*Vulpes vulpes*), goshawks (*Accipiter gentilis*), and great‐horned owls (*Bubo virginianus*) interact strongly with snowshoe hares as a prey resource (Feierabend & Kielland, 2015; Krebs, 2011). Most notable, however, is the obligate predator–prey relationship between Canada lynx (*Lynx canadensis*) and snowshoe hares (Elton & Nicholson, 1942; Ivan & Shenk, 2016; Krebs et al., 2001; Roth, Marshall, Murray, Nickerson, & Steury, 2007; Squires & Ruggiero, 2007). Canada lynx are designated as federally threatened in the contiguous 48 states of the United States (USFWS, 2000), as well as listed as threatened or endangered in some provinces of Canada (Poole, 2003). The conservation status of lynx highlights the political importance of conserving their main prey resource (e.g., USFWS, 2013), snowshoe hares. In order to effectively conserve snowshoe hares, however, forest managers need a detailed understanding of their habitat relationships across ecologically and management‐relevant scales. This is particularly true as the future of northern forests anticipates changes in the form of reduced snow (e.g., Klos, Link, & Abatzoglou, 2014; McKelvey et al., 2011) and increased wildfire (Liu, Goodrick, & Stanturf, 2013; Stavros, Abatzoglou, McKenzie, & Larkin, 2014), both of which significantly influence snowshoe hares (e.g., Cheng, Hodges, & Mills, 2015; Hodson, Fortin, & Bélanger, 2011; Mills et al. 2013; Sultaire, Pauli, Martin, Meyer, & Zuckerberg, 2016; Sultaire, Pauli, Martin, Meyer, Notaro, et al., 2016) .

Multiple studies have assessed habitat relationships of snowshoe hares using either occupancy, intensity of use, or density as the response of interest. For example, Thornton, Wirsing, Roth, and Murray (2013) documented a positive relationship between snowshoe hare occupancy and sites with more vegetation structure at the local and neighborhood scale. Allard‐Duchêne, Pothier, Dupuch, and Fortin (2014) identified a peak in browsing intensity by snowshoe hares in mid‐successional forests coincident with a peak in vegetation cover. Finally, Berg, Gese, Squires, and Aubry (2012) found that snowshoe hares were more abundant in late seral, multistoried forests with dense horizontal cover. This sample of studies illustrates a broader synthesis that vegetation structure rather than composition, and particularly dense horizontal cover at the stand level, largely influences habitat use and density of snowshoe hares (e.g., Fuller & Harrison, 2013; Griffin & Mills, 2009; Hodges, 2000a, 2000b; Hodges, Mills, & Murphy, 2009; Hodson, Fortin, & Bélanger, 2010; Hodson et al., 2011; Homyack, Harrison, & Krohn, 2007; Lewis, Hodges, Koehler, & Mills, 2011; Litvaitis, Sherburne, & Bissonette, 1985; Pietz & Tester, 1983; Sultaire, Pauli, Martin, Meyer, & Zuckerberg, 2016; Sultaire, Pauli, Martin, Meyer, Notaro, et al., 2016). In addition, landscape pattern can be important for snowshoe hare habitat use and density, especially in the fragmented landscapes within the southern part of their distribution (Griffin & Mills, 2009; Sultaire, Pauli, Martin, Meyer, & Zuckerberg, 2016; Sultaire, Pauli, Martin, Meyer, Notaro, et al., 2016; Thornton et al., 2013; Wirsing, Steury, & Murray, 2002). Griffin and Mills (2009) provided compelling evidence that open habitats, in a matrix of dense habitat, serve as population sinks, and an additional study found negative relationships between hare densities and amount of open habitats in close proximity to a focal patch (Lewis et al., 2011). Indeed, dense vegetation appears to be an essential resource for snowshoe hares throughout their distribution, primarily because of high predation rates (e.g., Feierabend & Kielland, 2015).

The previous studies of snowshoe hare habitat relationships have advanced our understanding and informed conservation efforts; however, many have exhibited limitations that restricted their inference. First, most assessments of snowshoe hare habitat have occurred at the local or patch scale. Thornton et al. (2013) also highlighted this limitation and indicated that future studies must extend beyond assessing only local factors. Second, the majority of previous studies have focused on one response (e.g., occupancy, intensity of use, or density), rather than jointly assessing two or more. Ecologists have demonstrated that biotic and abiotic factors influence occupancy and density differently (e.g., Boulangeat, Gravel, & Thuiller, 2012; Holbrook et al., 2016); thus, jointly evaluating multiple responses would provide a more complete understanding of snowshoe hare habitat. Finally, many of the previous studies used type‐based approaches for sampling (e.g., sampled predefined forest types) or analyses (e.g., used categorical forest maps). Type‐based approaches were historically the standard in ecology, but more recently researchers have emphasized the importance of maintaining the continuous nature of environmental gradients when possible and appropriate (Cushman, Gutzweiler, Evans, & McGarigal, 2010; Evans & Cushman, 2009; McGarigal, Tagil, & Cushman, 2009). The forests of the northern Rocky Mountains, USA (hereafter, Northern Rockies), exhibit substantial compositional and structural gradients, and thus, characterizing snowshoe hare habitat within a gradient‐based framework would advance our understanding of their habitat relationships. Furthermore, implementing a gradient‐based approach allows for broader application by forest managers because it circumvents issues related to the stability of predefined types as well as errors in stand delineation (Evans & Cushman, 2009).

**Figure 1:**
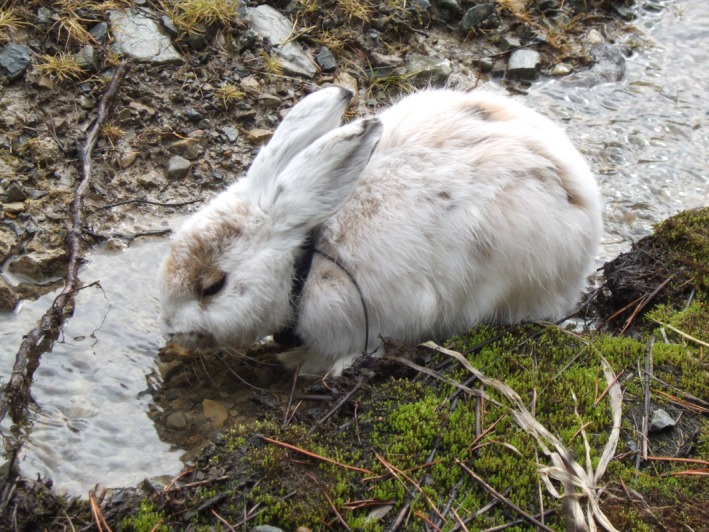
Snowshoe hare (*Lepus americanus*) in northwestern Montana, USA. Author of photograph: Laura Ehlen

Our objective for this study was to assess habitat relationships of snowshoe hares (Figure 1) in the Northern Rockies, using a multiscale and gradient‐based framework, with the goal of providing a more complete characterization of snowshoe hare habitat that could be used by forest managers. Specifically, we (1) assessed the relationship between horizontal cover and snowshoe hare occupancy and intensity of use at multiple scales, (2) estimated how forest metrics at the plot scale varied across the gradient of snowshoe hare use and the gradient of horizontal cover, and (3) modeled and mapped occupancy and intensity of use by snowshoe hares at the landscape scale. In addition, to provide conservation direction, we used our spatial maps to assess how the distribution and habitat use of snowshoe hares was related to protected (e.g., wilderness areas and national parks) versus multiple‐use lands (e.g., US National Forests). This work is novel in that (to our knowledge) it is the first study of snowshoe hares to (1) adopt a gradient‐based (vs. type‐based) approach concerning forest composition and structure, (2) implement a multiscale assessment for both occupancy and intensity of use, and (3) provide a model‐based map of predicted occupancy and intensity of use by snowshoe hares across a diverse land ownership. More broadly, however, this work advances our understanding of the habitat relationships of an essential herbivore that many iconic predators in the forests of North America rely upon (Krebs, 2011).

## 2 Materials and Methods

### 2.1 Study area

**Figure 2:**
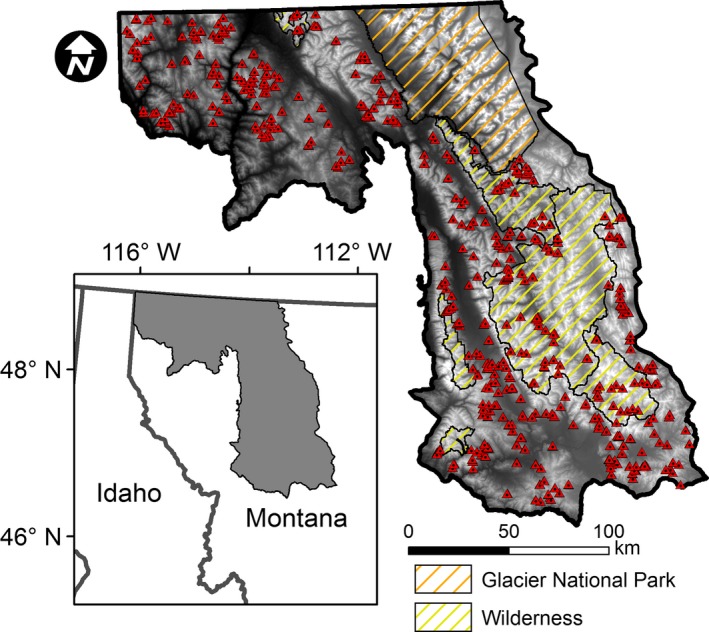
Distribution of plots (i.e., triangles) sampled during summer 2013 in northwestern Montana, USA. Inset shows our study area in relation to Montana and Idaho, USA

Our study area occurred in the Northern Rockies of northwestern Montana, USA, within the putative distribution of Canada lynx (Squires et al., 2013), which covered approximately 3.6 million ha (Figure 2). The study area followed natural topographic and vegetative boundaries and encompassed private lands and federal and state lands managed under a multiple‐use framework. In addition, multiple wilderness areas and Glacier National Park occur within our study area (Figure 2). This region is characterized as mixed conifer forests and ranges from 500 to nearly 3,200 m in elevation. These complex forests were composed of mostly ponderosa pine (*Pinus ponderosa*) and Douglas‐fir (*Pseudotsuga menziesii*) in lower elevations and lodgepole pine (*Pinus contorta*), western larch (*Larix occidentalis*), subalpine fir (*Abies lasiocarpa*) and Engelmann spruce (*Picea engelmannii*) at higher elevations.

### 2.2 Plot‐scale sampling

We randomly distributed and sampled >1,000 plots (20 × 20 m in size) throughout accessible locations within our study area during summer 2013 (Figure 2). At each plot, we measured percentage canopy cover (i.e., the upper canopy only) by species along a 5 × 5 m grid oriented in a north–south direction using a vertically projected moosehorn reading (25 readings of presence or absence/plot; Fiala, Garman, & Gray, 2006) for all trees ≥2.54 cm in diameter at 1.37 m above the ground (see Savage, Lawrence, & Squires, 2015). The dominant species recorded from canopy cover measurements included Douglas‐fir, lodgepole pine, western larch, Engelmann spruce, and subalpine fir. From the center of our sampling grid, we measured horizontal cover (which is highly associated with density of small‐diameter trees in the understory; see Squires, DeCesare, Kolbe, & Ruggiero, 2010) at 10 m in each of the four cardinal directions using a 2 m tall × 0.50 m wide coverboard divided into four 0.50‐m^2^ blocks (i.e., 16 readings/plot). Additionally, to accurately and efficiently sample trees ≥12.7 cm in diameter at 1.37 m above the ground (DBH), at the plot center we performed a Bitterlich variable radius plot, or prism plot, using a 10 basal area factor wedge prism (Lindsey, Barton, & Miles, 1958). We scanned 360° and recorded the DBH as well as species of all trees that were included in the plot. Data from variable radius plots overestimate tree density for trees <12.7 cm in DBH, so we restricted our sample to trees ≥12.7 cm in DBH and used measures of horizontal cover to index small‐tree density.

We sampled snowshoe hares by enumerating snowshoe hare pellets within five 5.1 × 305 cm subplots (Krebs, Gilbert, Boutin, & Boonstra, 1987; Krebs, Boonstra et al., 2001) placed at plot center and the four edges of our 20 × 20 m plot. These pellet counts were on uncleared plots and provided our metric of snowshoe hare occupancy (≥1 pellet) and intensity of use (i.e., mean number of pellets/subplot). Many studies have confirmed the positive relationship between snowshoe hare density and pellet counts (e.g., Berg & Gese, 2010; Krebs et al., 1987; Krebs, Boonstra et al., 2001; Mills et al., 2005; Murray, Ellsworth, & Zack, 2005); thus, it was conservative and appropriate to use pellets as a measure of intensity of use. We visited our sampling locations only once; therefore, we did not estimate detection probabilities for occupancy (sensu MacKenzie et al., 2002). However, it is unlikely that the detection probability was substantially less than one because we surveyed small and spatially defined areas (5.1 × 305 cm subplots) for snowshoe hare pellets rather than surveying for the animal itself. Because of the small area we surveyed within each 20 × 20 m plot, we interpreted our measure of snowshoe hare occupancy as conservative (i.e., false positives < false negatives). Finally, we assumed that potential differences in decay rates of pellets among habitats (e.g., Prugh & Krebs, 2004) was a relatively random source of error given the spatial extent of our analysis (i.e., 3.6 million ha).

### 2.3 Landscape‐scale environmental data

We considered a suite of vegetative, topographic, and climate variables, which we hypothesized would influence snowshoe hare occupancy and intensity of use (Table 1). We used percentage canopy cover for five conifer species (from Savage et al., 2015): lodgepole pine, western larch, Douglas‐fir, subalpine fir, and Engelmann spruce. Previous work indicated a positive relationship between snowshoe hares and spruce‐fir habitats (e.g., Berg et al., 2012; Ivan, White, & Shenk, 2014), and thus, we added a spruce‐fir variable to our suite. Second, we used Landfire Existing Vegetation Cover (EVC; LANDFIRE, 2012) data to calculate proportion of nonforested areas (i.e., naturally open, burns, timber harvests) as well as edge density, both of which could exhibit a negative or parabolic relationship with snowshoe hare occupancy and intensity of use (e.g., Griffin & Mills, 2009; Lewis et al., 2011; Thornton et al., 2013). Proportion of nonforest (nonforest includes open areas as well as sparse trees) and edge density were both based on a forest versus nonforest classification, which we defined as <40% canopy cover of trees within the Landfire EVC dataset. Photointerpretation confirmed that this decision was the most appropriate boundary for dense forest versus sparse to nonforest (we assessed multiple thresholds between 30 and 60%). We also used the potential vegetation groupings compiled by Region 1 of the USDA Forest Service (Milburn, Bollenbacker, Manning, & Bush, 2015) to calculate the proportion of area within cool and wet spruce‐fir potential vegetation types. Finally, we used Landsat TM images from summer 2011 (see Savage et al., 2015 for image processing details) to calculate the Normalized Difference Vegetation Index (Pettorelli et al., 2005) as well as the tasseled cap vegetation indices (Huang, Wylie, Yang, Homer, & Zylstra, 2002). Tasseled cap metrics include brightness, greenness, and wetness, which indicate soil reflectivity, amount of green vegetation, and amount of soil and vegetation moisture, respectively. We generally expected a positive relationship between green vegetation and snowshoe hares and a negative relationship between snowshoe hares and particularly open and wet locations (i.e., high values of brightness and wetness).

**Table 1:** Landscape covariates used in Random Forest models to assess occupancy and intensity of use of snowshoe hares (*Lepus americanus*) in western Montana, USA. Covariate codes ABLA, LAOC, PICO, PIEN, and PSME indicate subalpine fir (*Abies lasiocarpa*), western larch (*Larix occidentalis*), lodgepole pine (*Pinus contorta*), Engelmann spruce (*Picea engelmannii*), and Douglas‐fir (*Pseudotsuga menziesii*), respectively. NA indicates that the variable was removed due to colinearity (|*r*| > .75), and NS indicates that the covariate was not selected in the top model

Theme	Variable	Resolution^a^	Importance rank^b^	Units	Reference
Vegetation	PICO canopy cover	1,000, 500 m	7, 1	%	Savage et al. (2015)
	PIEN canopy cover	NA, NA	NA, NA	%	Savage et al. (2015)
	ABLA canopy cover	NA, NA	NA, NA	%	Savage et al. (2015)
	PIEN‐ABLA canopy cover	500, 250 m	12, 10	%	Savage et al. (2015)
	LAOC canopy cover	1,000, 1,000 m	2, 2	%	Savage et al. (2015)
	PSME canopy cover	1,000, 1,000 m	9, 6	%	Savage et al. (2015)
	Horizontal cover	250, 250 m	5, 3	%	This study
	Proportion of nonforest (<40% canopy cover equaled nonforest)	500, 250 m	6, 9	Proportion	Landfire EVC (2012)
	Forest edge density (40–100% canopy cover equaled patches)	500, 1,000 m	13, NS	m/m^2^	Landfire EVC (2012)
Vegetation indices	Normalized difference vegetation index (NDVI)	250, NA	NS, NA	Index	Pettorelli et al. (2005)
	Tasseled cap brightness	500, 250 m	8, 5	Index	Huang et al. (2002)
	Tasseled cap greenness	1,000, 1,000 m	4, 4	Index	Huang et al. (2002)
	Tasseled cap wetness	500, 250 m	3, 8	Index	Huang et al. (2002)
	Proportion of cool PIEN‐ABLA potential vegetation types	1,000, 250 m	10, NS	Proportion	Milburn et al. (2015)
Climate	Mean snow depth on 1 April 2012–2013^c^	1,000 m	1, 7	m	NOHRSC (2004)
Topography	Slope	NA, NA	NA, NA	Degrees	USGS
	Roughness	1,000, 250 m	11, NS	Index	Jenness (2004)
	Heat load index	250, 1,000 m	NS, NS	Index	McCune and Keon (2002)
	Compound topography index	250, 500 m	NS, NS	Index	Gessler et al. (1995)
	Topographic position index	500, 250 m	NS, NS	Index	Guisan et al. (1999)

a Resolution indicates the scale for a particular covariate that was selected for occupancy and intensity of use models, respectively.

b Importance rank was based on the mean decrease in accuracy for models of occupancy and intensity of use.

c We did not assess multiple scales because it was already at the coarsest resolution (1,000 m).

To characterize topography, we calculated derivatives of a digital elevation model (National Elevation Dataset, USGS). We computed slope, topographic roughness (Jenness, 2004), a heat load index (McCune & Keon, 2002), a topographic position index (Guisan, Weiss, & Weiss, 1999), and a compound topography index (Gessler, Moore, McKenzie, & Ryan, 1995). Our heat load index, topographic position index, and compound topography index represented hot‐dry to cool‐moist areas, relative concavity or convexity, and amount of water accumulation, respectively. We expected snowshoe hares to be associated with cool‐moist areas with intermediate water accumulation in the context of concave topographic locations (e.g., basins vs. ridges). All topographic metrics were calculated using either DEM Surface Tools for ArcGIS (Jenness, 2013) or Geomorphometric and Gradient Metrics Toolbox (Evans, Oakleaf, Cushman, & Theobald, 2014) within ArcGIS (ESRI, 2011).

Lastly, because of the relationship between snowshoe hares and snow (e.g., Mills et al. 2013, Sultaire, Pauli, Martin, Meyer, & Zuckerberg, 2016; Sultaire, Pauli, Martin, Meyer, Notaro, et al., 2016), coupled with the sensitivity of snow depth and extent to warming temperatures (Barnett, Adam, & Lettenmaier, 2005), we gathered spatially explicit data for snow depth from the Snow Data Assimilation System (SNODAS) within the National Operational Hydrologic Remote Sensing Center (NOHRSC, 2004). Previous analyses indicated a strong association between SNODAS‐derived estimates of snow depth and field measurements in the forested systems of the Northern Rockies (Clow, Nanus, Verdin, & Schmidt, 2012). We downloaded 1 April snow depth for 2012–2013 and averaged across years to produce a mean estimate for our study area. We expected a parabolic relationship between snow depth and snowshoe hares because hares occupy subalpine environments (vs. high‐elevation alpine areas with deeper snow, and low‐elevation areas with little snow; elevation‐snow depth *r *=* *.70) in the Northern Rockies.

#### 2.3.1 Horizontal cover mapping

Nearly all previous studies of snowshoe hare habitat relationships indicated a strong relationship with horizontal cover (Hodges, 2000a, 2000b). Thus, we sought to develop a new spatial map explicitly characterizing horizontal cover, which we could then use in our landscape‐level modeling of snowshoe hares (see Section “2.5”). Previous analyses within our study area indicated that horizontal cover is strongly associated with subalpine fir (Squires et al., 2010). Consequently, as spatial covariates for our model to map horizontal cover, we used the species‐specific canopy cover layers for the five dominant conifers within our study area (Savage et al., 2015), along with a stack of 22 Landsat bands and derived spectral components and 2 NAIP texture images (see Savage et al., 2015 for stack details), and four topographic metrics. The topographic metrics included a digital elevation model (National Elevation Dataset, USGS), slope, heat load index (McCune & Keon, 2002), and a compound topographic index (Gessler et al., 1995).

We built a Random Forest (RF) model (Breiman, 2001; Cutler et al., 2007) to evaluate our ability to spatially predict horizontal cover across our study area. Our sample size was 1,275 plots with a 70%–30% allocation among training and testing data, respectively. We used 1,275 because, based on photointerpretation, they were homogenous with respect to forest vegetation and thus captured a comparatively clean spectral signature. We evaluated model performance by testing whether the difference between observed and predicted values (from our testing data) equaled zero, and also assessed how much of the variation was explained (i.e., *R* ^2^) using linear regression. In addition, we assessed partial dependence plots from our RF model (Cutler et al., 2007) to understand what covariates were positively and negatively related to horizontal cover. All analyses were conducted in R (R Core Team, 2015) using the “randomForest” and “caret” packages (Kuhn et al., 2016; Liaw & Wiener, 2002).

**Figure 3:**
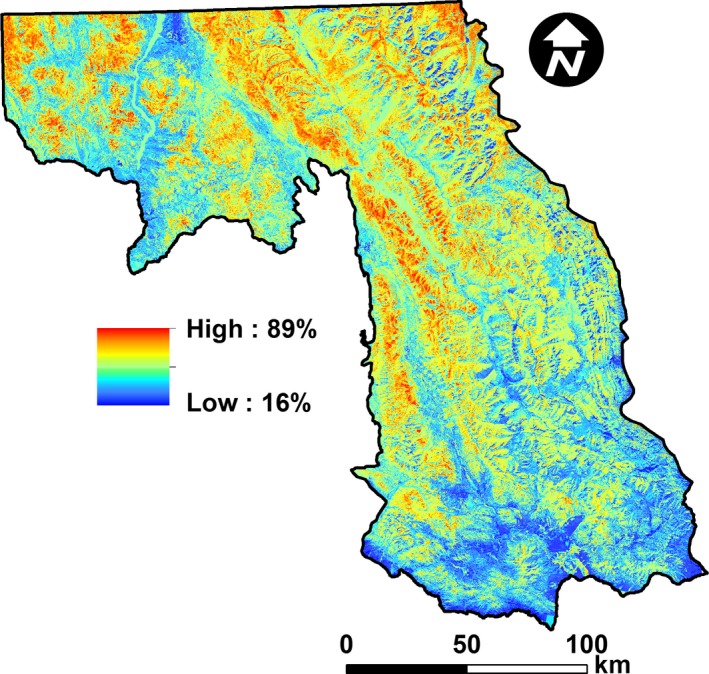
Predicted horizontal cover throughout our study area in northwestern Montana, USA

Partial dependence plots indicated that horizontal cover was most positively related to canopy cover of subalpine fir (consistent with Squires et al., 2010) and near‐infrared bands, and negatively related to the thermal infrared bands. Validation results on our testing data indicated no statistical difference between the observed and predicted values (*t*‐value = 0.74, *p = *.46, *df *=* *382), and a positive relationship between our observed and predicted values (slope* *=* *1.22, *t*‐value =* *11.47, *p *<* *.01, *df *=* *381). However, our model explained a modest 26% of the variation in horizontal cover (*R* ^2^ **=* *0.26) at the 30‐m resolution. To further assess our ability to explain horizontal cover, we implemented a scaling‐up procedure. We buffered all plots (i.e., 1,275) by 150 m (which was based on visual inspection in GIS), and if any buffers overlapped, we combined them into a single polygon. We then averaged our field measurements of horizontal cover and calculated the average predicted value across 30‐m cells within each polygon. Using linear regression, we documented a strong relationship (slope* *=* *1.45, *t‐*value = 19.60, *p *<* *.01, *df *=* *400) that explained approximately 50% of the variation in horizontal cover (*R* ^2^ = 0.49). These results suggest that we were successful in characterizing a horizontal cover gradient (particularly at broader resolutions), and thus, we generated a map of horizontal cover throughout our study area (Figure 3). Our map of horizontal cover served as one of our covariates for the landscape‐level modeling of habitat use by snowshoe hares (Table 1), which we expected to be strongly influential. We used the “raster” package (Hijmans, 2015) in R (R Core Team, 2015) as well as ArcGIS (ESRI, 2011) to assess our predictions of horizontal cover.

### 2.4 Plot‐scale analyses

To address our first objective, we modeled the effect of horizontal cover on snowshoe hare occupancy (*n *=* *1,297 for plots surveyed for snowshoe hares with associated forest structure data) and intensity of use (*n *=* *865 for plots with ≥1 snowshoe hare pellet and associated forest structure data) using logistic and quantile regression (Koenker & Bassett, 1978), respectively. Quantile regression is useful for characterizing the functional relationship between covariates across portions of the response distribution (see Brennan, Cross, & Creel, 2015; Cade & Noon, 2003). For instance, Cade and Noon (2003) demonstrated that the positive effect of shrub cover on pronghorn (*Antilocapra americana*) density increased with increasing pronghorn density. In other words, shrub cover was strongly linked to high pronghorn density, but other factors were contributing to the variation at moderate to low densities. We estimated the effect of horizontal cover across eight quantiles along the distribution of use by snowshoe hares (τ* *= 0.35, 0.45, 0.50, 0.55, 0.65, 0.75, 0.85, 0.95).

To satisfy our second objective, we calculated and compared means (i.e., assessed overlap among 95% CIs) for forest structure metrics (including only trees ≥12.7 cm in DBH) across the distribution of snowshoe hare use and horizontal cover. These analyses provided management‐relevant insight as to how forest structure varies across the gradient of snowshoe hare use and horizontal cover, a significant component of habitat for snowshoe hares. We grouped intensity of use and horizontal cover into five classes based on quantiles of their respective distributions (i.e., τ = 0.10, 0.30, 0.50, 0.70, 0.90) while maintaining relatively large sample sizes within each class (e.g., ≥157 observations/quantile). We used our field estimates of horizontal and canopy cover, but used the Forest Vegetation Simulator (FVS; Crookston & Dixon, 2005) to calculate overall and species‐specific tree density, basal area, quadratic mean diameter, basal area‐weighted DBH, and mean tree height from the data acquired at our prism plots. In addition, we used FVS to calculate overall tree density within three size classes of trees (e.g., 12.7–25.4 cm, 25.4–50.8 cm, 50.8–137.16 cm in DBH). All plot‐level analyses were conducted using the “quantreg” (Koenker, 2016), “Rmisc” (Hope, 2013), and “ggplot2” (Wickham, 2009) packages in R (R Core Team, 2015).

### 2.5 Landscape‐scale analyses

We built RF models (Breiman, 2001; Cutler et al., 2007) at the landscape scale to understand and predict snowshoe hare occupancy and intensity of use. Similar to previous studies (e.g., Boulangeat et al., 2012), we implemented RF as a classification for occupancy (*n *=* *1,344 for plots with snowshoe hare data) and regression for intensity of use based on occupied plots (*n *=* *898 for plots with ≥1 snowshoe hare pellet). Sample sizes were larger for our landscape‐scale analysis because we did not need forest structure data at the plot level (i.e., forest data were recorded on a subset of the 1,344 plots). We summarized landscape covariates across three scales between 6.25 and 100 ha (i.e., 250‐, 500‐, and 1,000‐m window) that captured home range sizes (e.g., ~6–10 ha; Hodges, 2000a) as well as annual displacement movements (<1,000 m; Griffin & Mills, 2009) of snowshoe hares. To begin our modeling process, we first ran separate RF models for each scale‐variant covariate (all except snow depth, which was only available at the coarsest scale evaluated; Table 1) to determine the most important scale as measured by the mean decrease in accuracy (Cutler et al., 2007). Next, we compiled all covariates at the most influential scale (Table 1) and removed those that were collinear (|*r*| > .75; Table 1). We then built our RF model with 5,000 bootstrapped trees and implemented a covariate selection procedure described by Murphy, Evans, and Storfer (2010), whereby we assessed the trade‐off in number of covariates and predictive ability across thresholds of model improvement ratios between 0.1–1 by 0.1 increments. We selected the model with the fewest covariates that maintained the highest predictive performance. We then assessed model fit of the classification model by reporting the “out‐of‐bag error” (OOBE), classification error among classes (i.e., unoccupied and occupied), and the predicted probabilities of occupancy for occupied and unoccupied plots. We assessed fit of the regression model using the RF *R* ^2^, root mean square error (RMSE), and assessing the correlation between observed and predicted values. For both models, we assessed model significance by randomizing the response (i.e., occupancy or intensity of use), calculating the OOBE or *R* ^2^ of each model (*n *=* *1,000), and determining whether the observed value (OOBE or *R* ^2^) from our built model was >95th percentile of the randomized values (*p *<* *.05), which indicates a significant model (Murphy et al., 2010). We then graphed the effects of our selected covariates on occupancy and intensity of use using partial dependence plots (Cutler et al., 2007). Finally, to help guide conservation efforts, we generated a predicted map of occupancy and intensity of use for snowshoe hares throughout our study area and assessed how both metrics were distributed across protected areas (i.e., Glacier National Park and wilderness; Figure 2) and multiple‐use lands. Specifically, we estimated the area of predicted occupancy and the mean predicted pellet density (i.e., pellets/5.1 × 305 cm subplots) within both areas. All landscape‐scale analyses were performed in ArcGIS (ESRI, 2011) or R (R Core Team 2015) using the “randomForest” (Liaw & Wiener, 2002), “rfUtilities” (Evans & Cushman, 2009), and “raster” (Hijmans, 2015) packages.

## 3 Results

Across our sample of plots, the number of trees ≥12.7 cm in DBH was distributed among Douglas‐fir (29%), lodgepole pine (20%), Engelmann spruce (15%), western larch (15%), and subalpine fir (14%); other species made up the remaining 8%. We determined that 67% of the plots were occupied by snowshoe hares as indexed by pellet counts (33% were unoccupied). We observed a range of 0–40.4 ($\overset{¯}{x}$ = 2.36) snowshoe hare pellets/5.1 × 305 cm subplot. For comparative purposes (e.g., Hodges et al., 2009; Mills et al., 2005), we applied the equation of Krebs, Boonstra et al. (2001) to the mean value within our categories of nonzero pellet densities (i.e., 0.2–1.2, 1.2–2.4, 2.4–5.2, and 5.2–40.4 pellets/subplot) to estimate a range of hare densities within our study area. Although our estimates might be biased high because our plots were not precleared, we nevertheless found that snowshoe hare densities were 0.28, 0.81, 1.48, and 4.21 hares/ha across our classes of pellet density, respectively. Our overall estimate (i.e., across all samples including zeros) was 1.01 snowshoe hares/ha.

### 3.1 Plot scale

**Figure 4:**
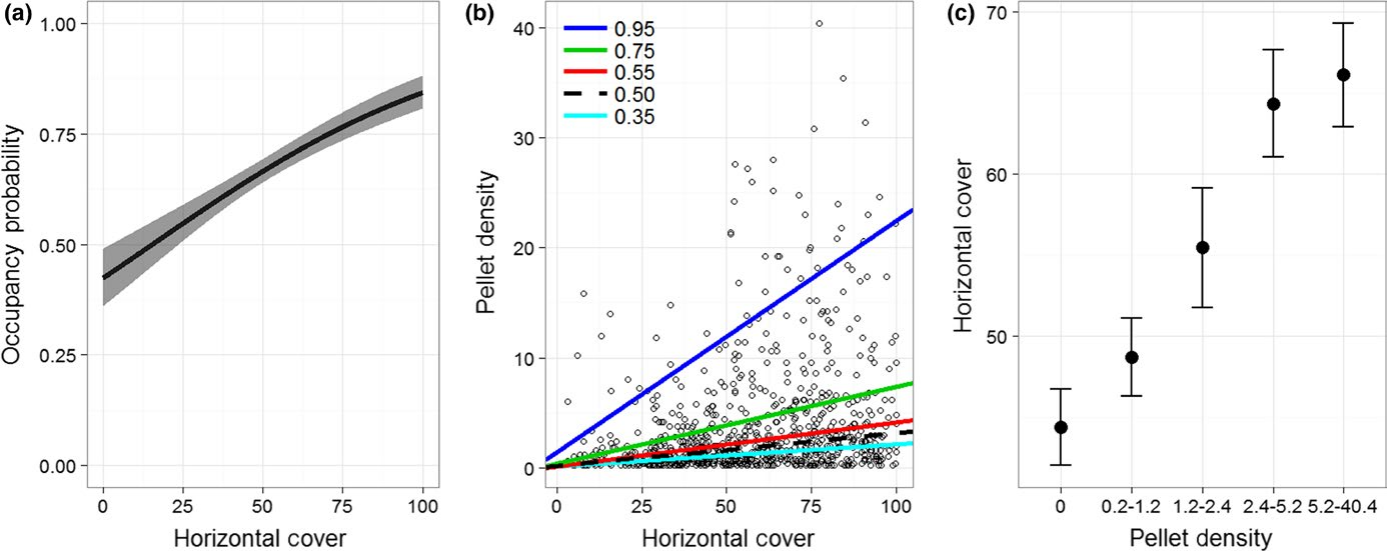
(a) Predicted occupancy (±95% CI) of snowshoe hares (*Lepus americanus*) as a function of horizontal cover. (b) Relationship between pellet density of snowshoe hares and horizontal cover for five quantiles of the pellet density distribution (τ * *=* * 0.35, 0.50, 0.55, 0.75, and 0.95). The black and dashed line indicates the median value (i.e., 0.50). (c) Mean (±95% CI) horizontal cover across the distribution of pellet densities

**Figure A1:**
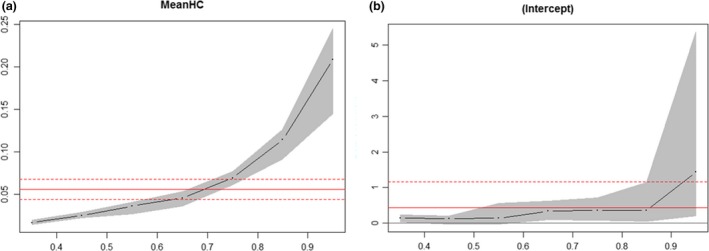
Estimated slope (a) and intercept (b) for quantile regression model assessing the effect of horizontal cover on intensity of use by snowshoe hares (*Lepus americanus*). We assessed eight quantiles between 0.35–0.95 by 0.10 increments. The solid red and dotted lines indicate median (i.e., 0.50) estimates and confidence bounds, respectively. The black line indicates the estimated slope (a) and intercept (b) as well as their respective error bounds. The slope differs from the median value, but the intercept does not, indicating that the pattern is more than a change in central tendency (Cade & Noon, 2003)

Occupancy and intensity of use by snowshoe hares were positively related to horizontal cover (Figure 4). Our logistic model indicated that the odds of snowshoe hare occupancy increased by 20% for every 10% increase in horizontal cover (*z*‐value* *=* *7.87, *df *=* *1,295, *p *<* *.001). Similarly, the effect of horizontal cover on intensity of use by snowshoe hares was statistically positive for all quantiles (all *p *<* *.05), but the slope increased with increasing quantiles (Figure A1 in Appendix A). The estimated intercept remained statistically similar across quantiles (Figure A1 in Appendix A), which indicated that the change in slope was more than simply a change in central tendency. Taken together, results from our quantile regression model provided evidence that not only was horizontal cover important for snowshoe hares, but that horizontal cover became increasingly important as use increased (Figure 4). Finally, estimates of horizontal cover increased with intensity of use by snowshoe hares, and exceeded 60% in areas used the most by snowshoe hares (Figure 4).

**Figure 5:**
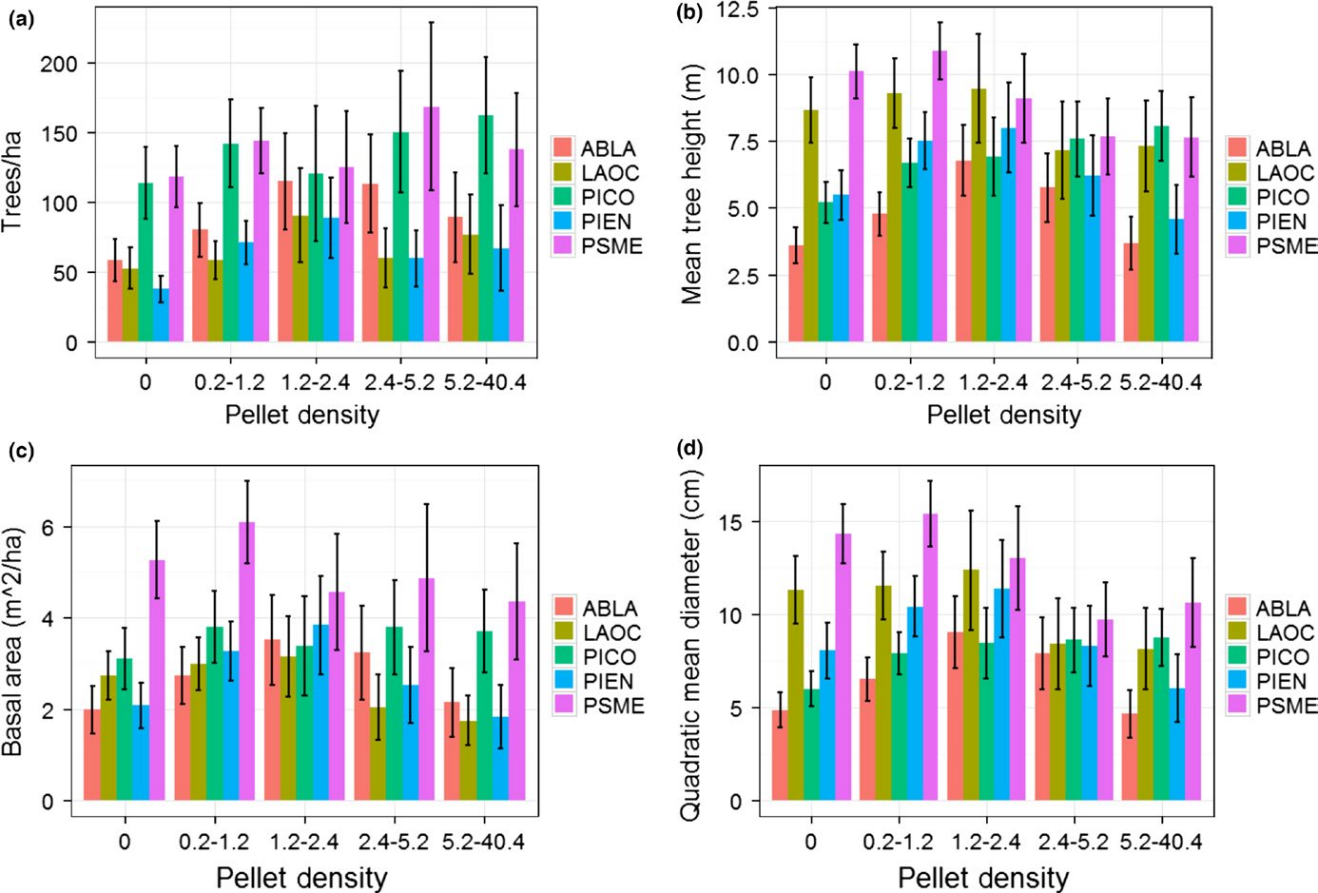
Mean (±95% CI) of trees/ha (a), tree height (b), basal area (c), and quadratic mean diameter (d) across the distribution of snowshoe hare (*Lepus americanus*) pellet densities. Codes ABLA, LAOC, PICO, PIEN, and PSME indicate subalpine fir (*Abies lasiocarpa*), western larch (*Larix occidentalis*), lodgepole pine (*Pinus contorta*), Engelmann spruce (*Picea engelmannii*), and Douglas‐fir (*Pseudotsuga menziesii*), respectively. All metrics were calculated using the Forest Vegetation Simulator (Crookston & Dixon, 2005) from data collected using a 10 basal area factor prism, and only included trees ≥12.7 cm (i.e., 5 inches) in DBH

**Figure 6:**
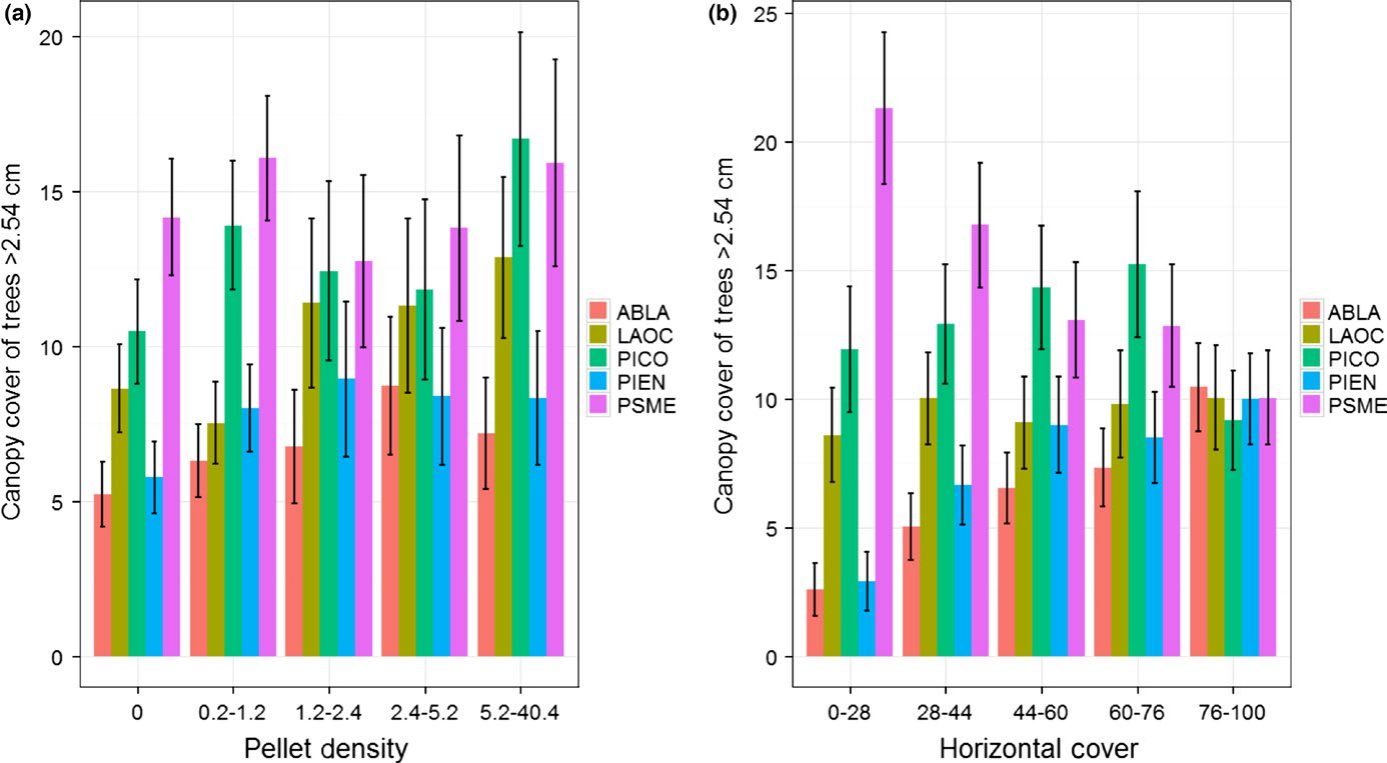
Mean (±95% CI) canopy cover across the distribution of snowshoe hare (*Lepus americanus*) pellet density (a) and horizontal cover (b). Codes ABLA, LAOC, PICO, PIEN, and PSME indicate subalpine fir (*Abies lasiocarpa*), western larch (*Larix occidentalis*), lodgepole pine (*Pinus contorta*), Engelmann spruce (*Picea engelmannii*), and Douglas‐fir (*Pseudotsuga menziesii*), respectively. We estimated percentage canopy cover (i.e., the upper canopy only) along a 5 × 5 m grid using a vertically projected moosehorn reading for trees ≥2.54 cm (i.e., 1 inch) in DBH

**Figure 7:**
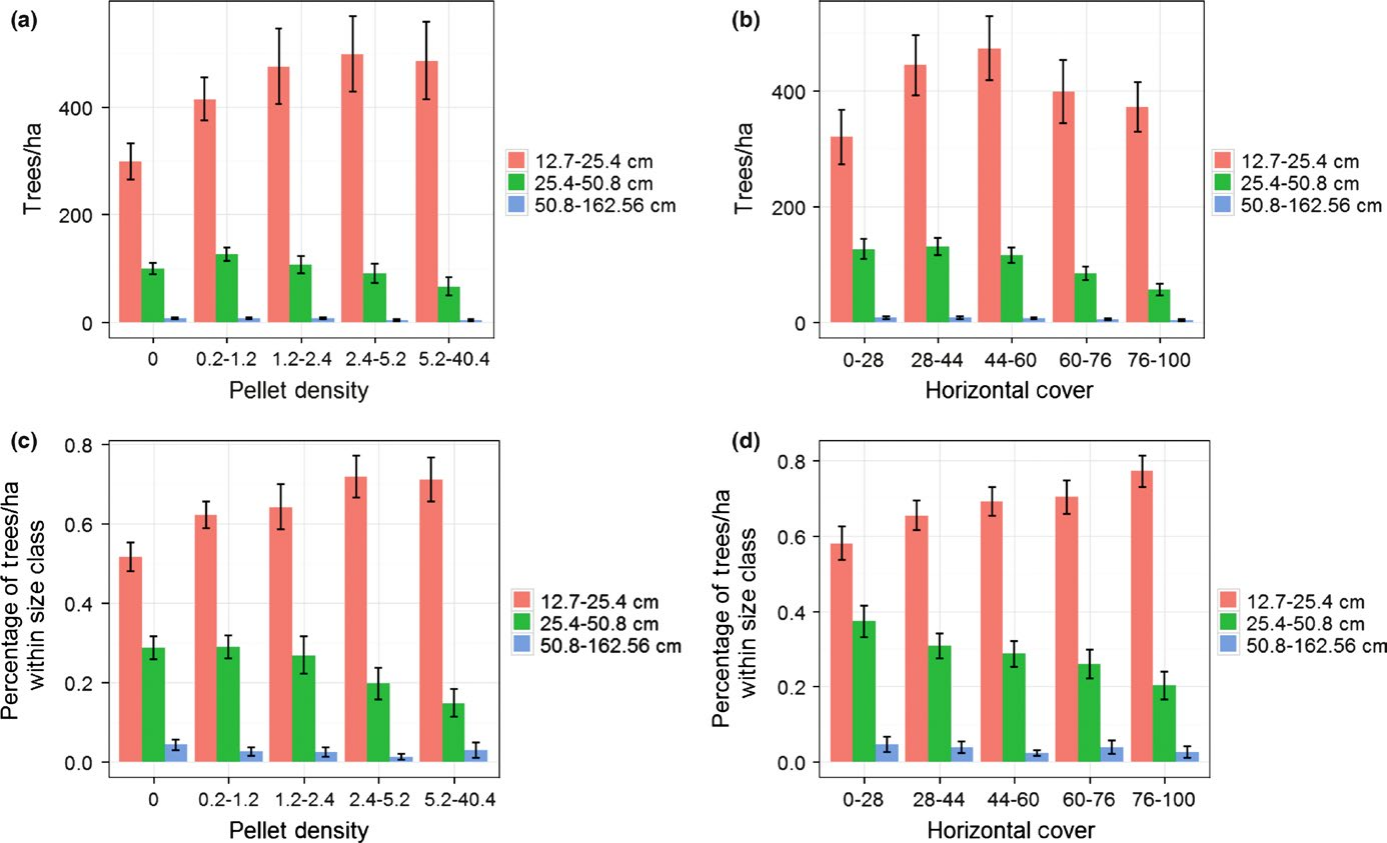
Mean (±95% CI) trees/ha within size classes across the distribution of snowshoe hare (*Lepus americanus*) pellet density (a) and horizontal cover (b). Mean percentage (± 95% CI) of trees/ha within size classes across the distribution of pellet density (c) and horizontal cover (d). All metrics were calculated using the Forest Vegetation Simulator (Crookston & Dixon, 2005) from data collected using a 10 basal area factor prism, and only included trees ≥12.7 cm (i.e., 5 inches) in DBH

Metrics of forest structure (i.e., for trees ≥12.7 cm in DBH) varied across intensity of use by snowshoe hares (see Table 2 and Table A1 in Appendix A, for summary of structural values), and to illustrate these changes, we focused on comparisons between the absent and highest use class. For all tree species, we observed no change in trees/ha (Figure 5). However, overall stem density (trees/ha) increased between the absent and highest use class, and the estimate for the highest use class was 556 trees/ha (95% CI* *=* *479–633; Table 2). We observed an increase in mean tree height and quadratic mean diameter for lodgepole pine, and a decrease for Douglas‐fir, between the absent and highest use class (Figure 5). Similarly, we observed a decrease in quadratic mean diameter for western larch (Figure 5). Tree height across all species exhibited no change along intensity of use by snowshoe hares, but the estimate for the highest use class was 15 m (95% CI* *=* *14–16; Table 2). In contrast, quadratic mean diameter decreased between absence and high use by snowshoe hares and was 18 cm (95% CI* *=* *16–19) for areas with the highest use (Table 2). Basal area decreased for western larch (Figure 5), but the overall basal area remained consistent across intensity of use and was 14.7 m^2^/ha (95% CI* *=* *12.7–16.6) for areas exhibiting the highest use by snowshoe hares (Table 2). An additional observation across the gradient of snowshoe hare pellet density was that subalpine fir and Engelmann spruce exhibited a parabolic pattern for trees/ha, tree height, basal area, as well as quadratic mean diameter (Figure 5), suggesting a positive relationship up to a threshold. Canopy cover increased for lodgepole pine and western larch (Figure 6), and the overall estimate increased with intensity of use by snowshoe hares and was 67% (95% CI* *=* *64–71) in areas used most (Table 2). Finally, we found an increase in trees/ha for the 12.7‐ to 25.4‐cm size class, and a decrease in the 25.4‐ to 50.8‐cm and 50.8‐ to 162.56‐cm size classes (Figure 7). The proportions of size classes across snowshoe hare use exhibited a similar pattern and highlighted that areas receiving the most use were forests with a substantial component of medium‐sized trees but also had multiple canopy layers (i.e., multistoried forests; Figure 7).

**Table 2:** Mean (and 95% CIs) forest structural metrics associated with the gradient of snowshoe hare (*Lepus americanus*) pellet density (pellet density* *=* *mean number of pellets/5.1 × 305 cm subplots) and the associated estimate of hare density (hares/ha) using the equation of Krebs, Boonstra et al. (2001). Codes BAWDBH, QMD, and DBH indicate basal area‐weighted diameter at breast height (__(tree basal area × DBH)/total basal area), quadratic mean diameter, and mean diameter at breast height. All metrics (except DBH and canopy cover) were calculated within the Forest Vegetation Simulator (FVS; Crookston & Dixon, 2005) from data collected using a 10 basal area factor prism, and only included trees ≥12.7 cm (i.e., 5 inches) in DBH. We calculated mean DBH of trees recorded with the 10 basal area factor prism, and estimated percentage canopy cover (i.e., the upper canopy only) along a 5 × 5 m grid using a vertically projected moosehorn reading. Both English and metric (italicized) units are provided and are as follows: basal area (ft^2^/acre, *m* ^*2*^ */ha*), tree density (trees/acre, *trees/ha*), BAWDBH (inches, *cm*), QMD (inches, *cm*), mean diameter at breast height (inches, *cm*), and mean tree height (feet, *m*)

Pellet density	Hares/ha	Sample size	Basal area	Tree density	BAWDBH	QMD	DBH	Mean tree height	Canopy cover (%)
None	0	432	71 (65*–*77)*16.3 (15.0–17.6)*	163 (148*–*179)*404 (365–442)*	10.1 (9.5*–*10.7)*25.7 (24.2–27.2)*	8.5 (8.1*–*9.0)*21.7 (20.5–22.9)*	10.1 (9.5*–*10.7)*25.6 (24.1–27.1)*	54 (52*–*57)*16.5 (15.8–17.3)*	52 (50*–*55)
0.2–1.2	0.28	378	91 (85*–*97)*20.9 (19.5–22.3)*	222 (205*–*238)*548 (507–589)*	10.4 (9.9*–*10.9)*26.4 (25.2–27.6)*	8.8 (8.4*–*9.2)*22.4 (21.4–23.4)*	10.4 (9.9*–*10.8)*26.3 (25.1–27.5)*	58 (56*–*60)*17.6 (17.0–18.2)*	61 (59*–*63)
1.2–2.4	0.81	158	89 (80*–*97)*20.4 (18.4–22.3)*	238 (209*–*267)*589 (518–660)*	10.0 (9.2*–*10.7)*25.3 (23.4–27.3)*	8.5 (7.9*–*9.1)*21.7 (20.2–23.2)*	9.9 (9.2*–*10.7)*25.2 (23.4–27.1)*	56 (53*–*59)*17.2 (16.3–18.1)*	64 (60*–*67)
2.4–5.2	1.48	157	78 (69*–*86)*17.8 (15.9–19.8)*	240 (211*–*270)*593 (520–666)*	8.7 (8.1*–*9.4)*22.2 (20.6–23.9)*	7.6 (7.1*–*8.1)*19.2 (17.9–20.5)*	8.7 (8.1*–*9.3)*22.1 (20.5–23.7)*	52 (49*–*55)*15.9 (15.0–16.7)*	62 (59*–*66)
5.2–40.4	4.21	172	64 (55*–*72)*14.7 (12.7–16.6)*	225 (194*–*256)*556 (479–633)*	8.1 (7.3*–*8.9)*20.6 (18.6–22.5)*	7.0 (6.4*–*7.7)*17.9 (16.4–19.4)*	8.0 (7.3*–*8.7)*20.4 (18.5–22.2)*	49 (46*–*53)*15.0 (14.1–16.0)*	67 (64*–*71)

**Figure 8:**
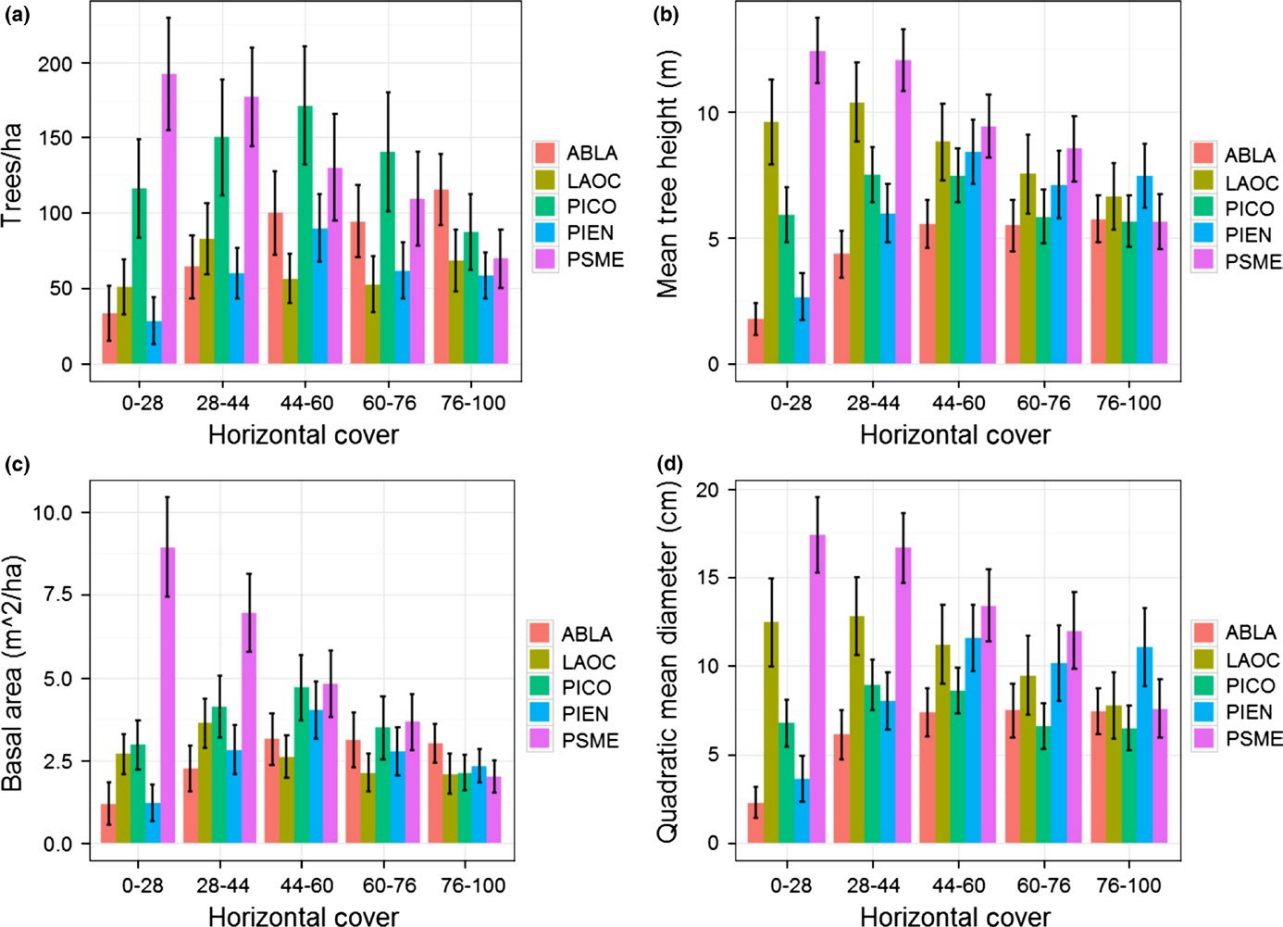
Mean (±95% CI) of trees/ha (a), tree height (b), basal area (c), and quadratic mean diameter (d) across the distribution of horizontal cover. Codes ABLA, LAOC, PICO, PIEN, and PSME indicate subalpine fir (*Abies lasiocarpa*), western larch (*Larix occidentalis*), lodgepole pine (*Pinus contorta*), Engelmann spruce (*Picea engelmannii*), and Douglas‐fir (*Pseudotsuga menziesii*), respectively. All metrics were calculated using the Forest Vegetation Simulator (Crookston & Dixon, 2005) from data collected using a 10 basal area factor prism, and only included trees ≥12.7 cm (i.e., 5 inches) in DBH

Similar to patterns within the snowshoe hare data, we observed changes in forest metrics (i.e., for trees ≥12.7 cm in DBH) across the gradient of horizontal cover. To highlight those changes, we focused on comparisons between the lowest and highest cover classes of horizontal cover. Subalpine fir and Engelmann spruce increased and Douglas‐fir decreased between the lowest and highest class for all forest metrics (i.e., trees/ha, tree height, basal area, quadratic mean diameter, and canopy cover; Figures 6 and 8). In addition, we observed a decrease in tree height and quadratic mean diameter for western larch (Figure 8) and found a parabolic relationship for trees/ha and basal area of lodgepole pine across the gradient of horizontal cover (Figure 8). We documented no other changes in forest metrics by conifer species (Figures 6 and 8). Lastly, we identified a pattern for horizontal cover similar to snowshoe hare use in terms of trees/ha across size classes (Figure 7), indicating that multistoried forests with a substantial component of medium‐sized trees are important for high horizontal cover.

### 3.2 Landscape scale

**Figure 9:**
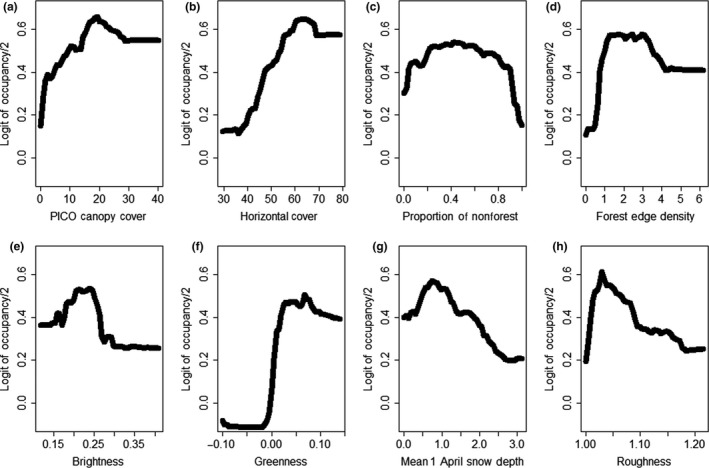
Partial dependence plots displaying the relationship between occupancy probability of snowshoe hares (*Lepus americanus*) and the covariates (a–h) that exhibited a consistent trend from our top Random Forest model. See Table 1 for covariate descriptions

Our best classification model characterizing occupancy of snowshoe hares contained 13 covariates (Table 1) and exhibited an OOBE of 25%, which was statistically less than random expectation (*p *<* *.001). Our OOBE was weighted toward our unoccupied class (unoccupied vs. occupied commission and omission error was 50 and 34% and 13 and 22%, respectively), indicating our model had difficulties assigning absence, but performed well when assigning presence. The average predicted probability of occupancy at occupied and unoccupied plots was 0.91 (range* *=* *0.54–0.99) and 0.18 (range* *=* *0.03–0.54), respectively. Snowshoe hare occupancy was positively related to canopy cover of lodgepole pine, horizontal cover, and tasseled cap greenness (Figure 9). In addition, occupancy exhibited a positive but quadratic relationship with proportion of nonforest, forest edge density, tasseled cap brightness, mean 1 April snow depth, topographic roughness, and tasseled cap wetness (Figure 9, Figure A1 in Appendix B). The remaining five covariates did not display a general and consistent trend with occupancy (Figure A1 in Appendix B).

**Figure 10:**
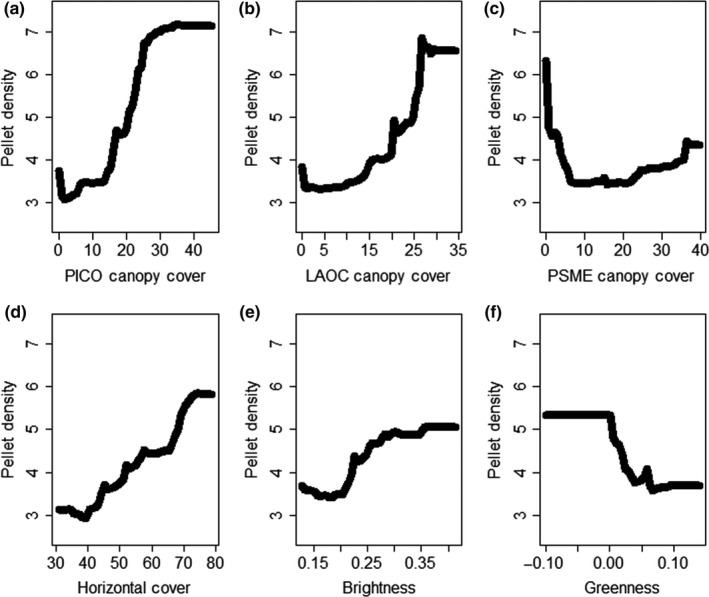
Partial dependence plots displaying the relationship between intensity of use by snowshoe hares (*Lepus americanus*) and the covariates (a–f) that exhibited a consistent trend from our top Random Forest model. See Table 1 for covariate descriptions

**Figure B2:**
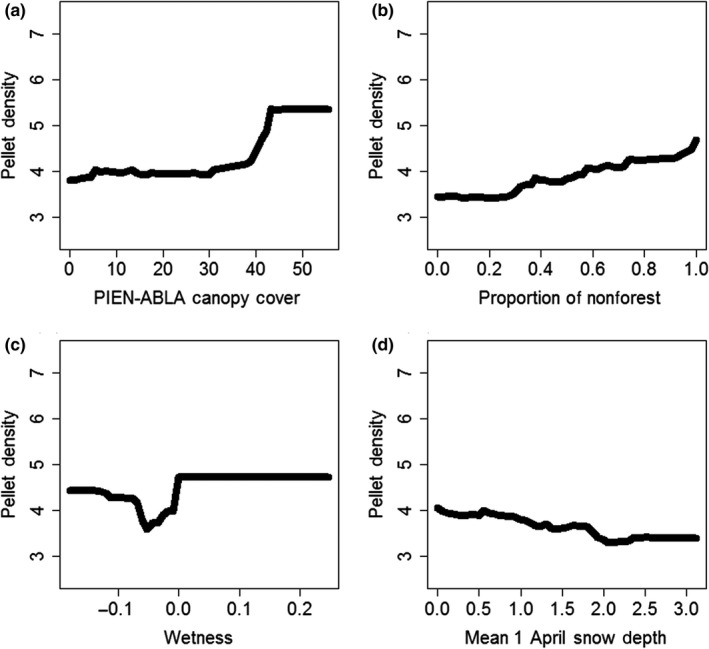
Partial dependence plots displaying the relationship between intensity of use by snowshoe hares (*Lepus americanus*) and covariates (a–d) that were included in our top Random Forest model, but did not exhibit a consistent trend. See Table 1 for covariate descriptions

The top regression model characterizing intensity of use by snowshoe hares contained 10 covariates (Table 1) and explained 32% of the variation in use (RF *R* ^2^ **=* *32%), which was statistically greater than random (*p *<* *.001). The RMSE for our top model was 4.22 and the correlation between observed and predicted values was high (*r *=* *.96). Intensity of use by snowshoe hares was positively related to canopy cover of lodgepole pine and western larch, as well as horizontal cover and tasseled cap brightness (Figure 10). Canopy cover of Douglas‐fir and tasseled cap greenness were negatively related to intensity of use (Figure 10). The remaining four covariates did not exhibit a consistent trend with intensity of use by snowshoe hares (Figure B2 in Appendix B).

**Figure 11:**
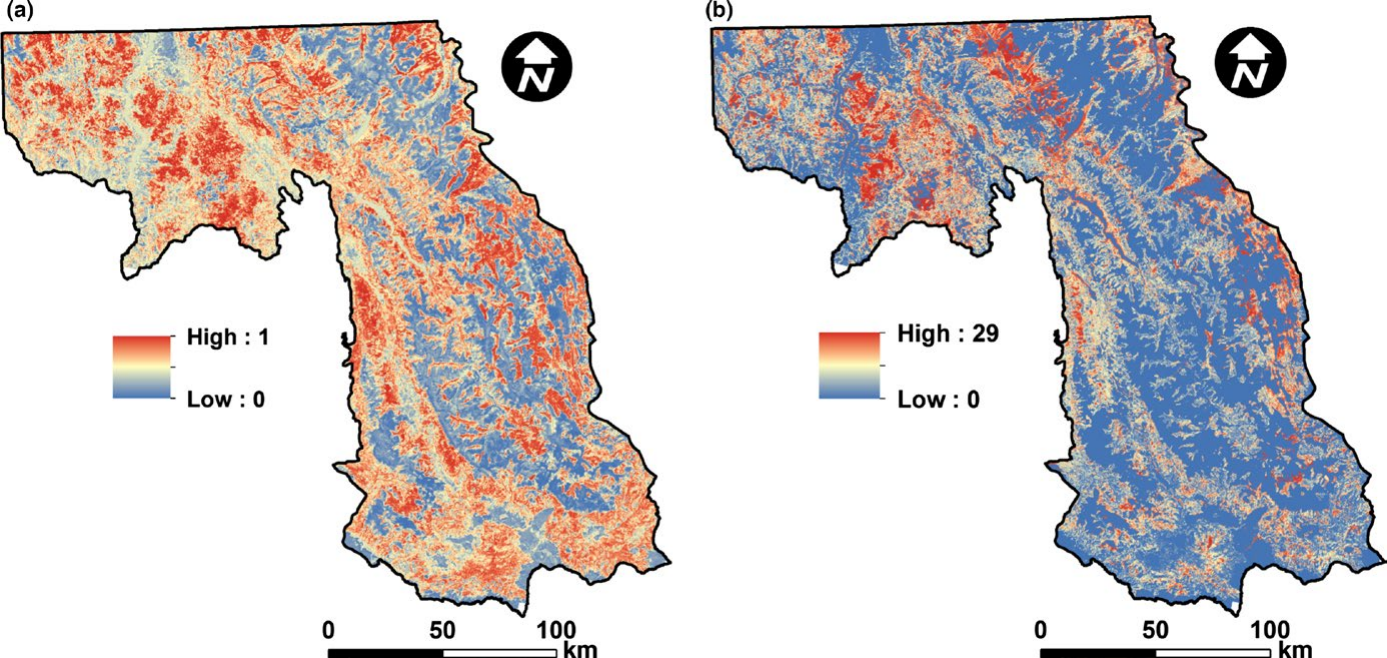
Predicted probability of occupancy (a) and intensity of use (b; indexed by the mean number of pellets/5.1 × 305 cm subplot) of snowshoe hares (*Lepus americanus*) throughout western Montana, USA. To account for absence, we multiplied our occupancy mask by the predicted intensity of use to produce our map of snowshoe hare intensity of use (b)

We used our top models characterizing occupancy and intensity of use by snowshoe hares to spatially map these responses across our study area (Figure 11). We found that the area of predicted occupancy and absence of snowshoe hares within Glacier National Park and wilderness areas was 4,032 km^2^ (37%) and 6,896 km^2^ (63%), respectively. The area of predicted occupancy and absence on multiple‐use lands was 14,868 km^2^ (59%) and 10,236 km^2^ (41%), respectively. These results suggested that protected areas captured more area of predicted absence, and less predicted presence, of snowshoe hares compared to multiple‐use lands. Similarly, the predicted pellet density was 1.26 pellets/subplot for protected areas relative to multiple‐use lands, which was 2.23 pellets/subplot.

## 4 Discussion

Snowshoe hares are an ecologically important herbivore and prey species in northern forests of North America (Krebs, 2011), and therefore, understanding their habitat relationships will help guide ecosystem‐level conservation and management. Because of our gradient and multiscale approach, we advanced the understanding of snowshoe hare habitat relationships on multiple levels. First, we demonstrated that both occupancy and intensity of use by snowshoe hares increased with horizontal cover, and highlighted that the influence of horizontal cover becomes stronger with increasing use (and likely density) of snowshoe hares. Second, our work indicated that subalpine fir and Engelmann spruce are the species that provide the high horizontal cover that is important for snowshoe hares, as well as identified a species‐specific association between hares and lodgepole pine across scales. Previous work has highlighted that lodgepole pine is more nutritious than other common conifers and that browsing by snowshoe hares is consistent with nutritional quality (Ellsworth, Wirsing, Shipley, & Murray, 2013). The association we documented between snowshoe hares and lodgepole pine provides support for the hypothesis that high‐quality nutrition substantively influences patterns of habitat use and that use is not simply driven by predation risk (e.g., Ellsworth et al., 2013; Hodges & Sinclair, 2003, 2005). In the mixed conifer forests of the Northern Rockies, the abundance of horizontal cover (e.g., subalpine fir and Engelmann spruce) and lodgepole pine, arranged in a multistoried and dense structure, appear to be the important aspects of habitat for snowshoe hares. Lastly, we observed a parabolic association between snow depth (positive between ~0.2–1 m) and occupancy of snowshoe hares, and snow depth ranked as our most important covariate characterizing occupancy (Table 1). Snow extent and, by extension, snow depth are projected to decrease within the Northern Rockies (e.g., Klos et al., 2014; McKelvey et al., 2011), which will likely have substantial implications for the distribution of snowshoe hares (e.g., Mills et al., 2013; Sultaire, Pauli, Martin, Meyer, & Zuckerberg, 2016; Sultaire, Pauli, Martin, Meyer, Notaro, et al., 2016; Zimova, Mills, & Nowak, 2016) as well as the predators that rely on them (e.g., Canada lynx). Collectively, our work provides a new, multiscale and gradient‐based lens on habitat relationships of snowshoe hares and offers specific insight for forest management and snowshoe hare conservation.

### 4.1 Plot‐scale patterns

Previous work has identified a positive relationship between snowshoe hare density and horizontal cover as well as spruce‐fir and lodgepole pine forests (Berg et al., 2012; Cheng et al., 2015; Hodges et al., 2009; Ivan et al., 2014; Koehler, 1990). Our data support these conclusions; however, the interpretation of our results is more nuanced. Our analyses highlighted that the abundance of spruce‐fir is more associated with horizontal cover than any other species of conifer (Figures 6 and 8), and we found that horizontal cover was strongly associated with occupancy and intensity of use by snowshoe hares (Figure 4). However, we did not discover any consistent relationship between snowshoe hare occupancy or intensity of use and spruce‐fir per se (although see parabolic relationships in Figure 5). Thus, our data suggest that horizontal cover is an important attribute influencing snowshoe hares and that this attribute is associated with forests that have a substantial spruce‐fir component (relative to those without a spruce‐fir component). Preserving the horizontal cover that spruce‐fir trees provide within the mixed conifer context of the Northern Rockies will likely be important for the conservation of snowshoe hares.

Moreover, we found a species‐specific association between snowshoe hares and abundance of lodgepole pine, which we attributed mostly to nutritional mechanisms. Ellsworth et al. (2013) discovered that lodgepole pine produced higher levels of digestible protein than other common conifers in the Northern Rockies (e.g., Douglas‐fir, subalpine fir, Engelmann spruce, western larch) and that overwinter depletion of biomass and browsing by snowshoe hares was most associated with lodgepole pine. Although our data indicate that areas with high use by snowshoe hares are indeed highly mixed conifer forests (e.g., Figure 5), forest managers within our study region could use lodgepole pine as well as spruce‐fir, or more specifically horizontal cover, as initial indicators of potential snowshoe hare habitat.

Our results reinforced previous studies highlighting the importance of dense forests for snowshoe hares (e.g., Berg et al., 2012; Griffin & Mills, 2007; Hodges et al., 2009; Hodson et al., 2011; Ivan et al., 2014; Lewis et al., 2011). Our data indicated that dense horizontal cover within multistoried forests with a substantial component of medium‐sized trees (i.e., 12.7–25.4 cm) produced the highest use by snowshoe hares, which was also found in previous studies within Montana (Griffin & Mills, 2007), Washington (Koehler, 1990; Lewis et al., 2011), Wyoming (Berg et al., 2012; Hodges et al., 2009), and Colorado (Ivan et al., 2014). Results from our data support conclusions similar to previous studies in that disturbing (e.g., cutting or burning) multistoried forests with high stem densities (particulary in the understory) would likely have a negative effect on snowshoe hares in the short term (e.g., Abele, Wirsing, & Murray, 2013; Griffin & Mills, 2007), but may benefit them in the future (e.g., 20–50 years; Hodson et al., 2011; Allard‐Duchêne et al., 2014).

### 4.2 Landscape‐scale patterns

At the landscape level, our study is the first to spatially map horizontal cover for modeling snowshoe hare habitat, as well as to model and map both occupancy and intensity of use of snowshoe hares (although see recent maps of occupancy in Sultaire, Pauli, Martin, Meyer, & Zuckerberg, 2016; Sultaire, Pauli, Martin, Meyer, Notaro, et al., 2016). Analyses of our maps indicated that protected areas (i.e., wilderness and Glacier National Park) captured less area of predicted occupancy of snowshoe hares than expected and that the predicted pellet density was also lower in protected areas relative to multiple‐use lands. This pattern is consistent with national parks disproportionately protecting alpine habitats (e.g., Bunn, 2009) and emphasizes the importance of multiple‐use lands (e.g., national forests, state and private lands) for the conservation of snowshoe hares and their predators within the Northern Rockies. In addition, our maps indicated that occupancy and intensity of use by snowshoe hares were patchily distributed at a course scale, which generally contrasts with previously developed maps in the north‐central continental USA (see occupancy maps in Sultaire, Pauli, Martin, Meyer, & Zuckerberg, 2016; Sultaire, Pauli, Martin, Meyer, Notaro, et al., 2016). The spatial products we provide in this study advance the landscape‐level understanding of snowshoe hares in the Northern Rockies, and also provide a basis of comparison for future modeling efforts assessing changes in the distribution and density of snowshoe hares. However, it is important to mention that subsequent analyses of our snowshoe hare maps should be at course resolutions (e.g., ≥100 m^2^), and we suggest caution when analyzing predicted values of snowshoe hare use because unmodeled temporal processes (e.g., predation, source–sink dynamics, cyclicity) could induce substantial variation. Our map of predicted occupancy, however, should be comparatively more stable because it approximates the realized Grinnellian niche (Grinnell, 1917; Hirzel & Le Lay, 2008). Developing these spatial products specifically within the Northern Rockies was important because this landscape is projected to experience substantial changes via reduction in snow (e.g., Klos et al., 2014; McKelvey et al., 2011) and increased wildfire (Liu et al., 2013; Stavros et al., 2014).

Of the few studies on snowshoe hares that have been conducted at a landscape level, results indicated that occupancy is positively associated with vegetation cover, snow cover, and surrounding population density (Sultaire, Pauli, Martin, Meyer, & Zuckerberg, 2016; Sultaire, Pauli, Martin, Meyer, Notaro, et al., 2016; Thornton et al., 2013). Furthermore, Lewis et al. (2011) highlighted that vegetation cover at the local and neighborhood level was important for density of snowshoe hares. Our occupancy results support the notion that vegetation cover and perhaps moisture content are important for snowshoe hares in that we observed a positive relationship between occupancy and canopy cover of lodgepole pine, horizontal cover, and tasseled cap greenness and wetness. Snow depth, however, was the most important variable characterizing occupancy of snowshoe hares, which supports the recent findings of Sultaire, Pauli, Martin, Meyer, & Zuckerberg (2016) and Sultaire, Pauli, Martin, Meyer, Notaro, et al. (2016) indicating that snow cover is more important than forest cover for characterizing snowshoe hare occupancy. In addition, we discovered that occupancy of snowshoe hares was associated with relatively flat topography, and perhaps some level of disturbance at a coarse resolution. Although canopy and horizontal cover are clearly important, our results suggest that some open areas (e.g., edges) within a matrix of high‐quality cover provide resources for increased use by snowshoe hares. Finally, similar to occupancy, the intensity of use by snowshoe hares exhibited a positive relationship with canopy cover (both lodgepole and western larch) and horizontal cover. We attributed the positive effect of lodgepole pine to similar nutritional mechanisms aforementioned (e.g., Ellsworth et al., 2013). However, the positive effect of western larch appeared to be related to a broad‐scale productivity gradient in that western larch tended to occur only in multiple‐use lands (vs. wilderness and national parks), and was most abundant in the northwestern portion of our study area (i.e., considered most productive). We observed a negative effect of Douglas‐fir on intensity of use by snowshoe hares, which was expected given the low level of horizontal cover associated with stands dominated by Douglas‐fir (Figures 6 and 8). The positive effect of tasseled cap brightness and a negative effect of greenness are consistent with the hypothesis that a few open areas (e.g., edges) within a matrix of high‐quality cover could provide additional foraging opportunities for snowshoe hares. This hypothesis is supported by previous work indicating that foraging behavior by snowshoe hares was largely associated with food supply (e.g., Ellsworth et al., 2013; Hodges & Sinclair, 2005), and not simply driven by predation risk.

### 4.3 Foreseeable conservation challenges

Our work also highlighted the foreseeable challenges facing decision makers that are related to climate‐induced reductions in snow depth and increases in wildfire. Evidence suggests that changes in snow extent and depth will continue to have direct effects on snowshoe hare distribution and abundance because of mismatches in coat color leading to increased mortality (e.g., Mills et al., 2013; Zimova et al., 2016). Similarly, the observed and projected increase in wildfire within the Northern Rockies could impact forest structure, composition, and landscape arrangement, all of which could certainly influence occupancy and abundance of snowshoe hares. For instance, Picotte, Peterson, Meier, and Howard (2016) demonstrated that subalpine habitats in the Rocky Mountains have exhibited temporal increases in both fire size and severity during 1984–2010, which together could act as a large‐scale homogenization process in terms of forest structure (e.g., more stand initiation and regeneration) and species composition (e.g., increase in fire‐adapted species such as lodgepole pine). Previous work has indicated a positive response of snowshoe hares to fire (e.g., Cheng et al., 2015; Hodson et al., 2011), but there is a temporal delay, suggesting that the unburned matrix surrounding fires might be critically important in the short term (e.g., Abele et al., 2013; Ausband & Baty, 2005; Lewis et al., 2011). These broad‐scale changes suggest that forest management for snowshoe hare habitat will likely be a nuanced and landscape‐level endeavor.

Additionally, perhaps of equal concern are the indirect effects associated with changes induced by snow reduction and increased wildfire. For example, predation is the main process driving population dynamics of snowshoe hares (Feierabend & Kielland, 2015; Krebs, 2011; Wirsing et al., 2002), and changes in snow, or vegetation structure due to fire, could introduce or remove predators. These indirect effects might be of more concern in the southern range of snowshoe hares because densities tend to be lower than the northern range and the landscapes are generally more fragmented (Hodges et al., 2009; Thornton et al., 2013; Wirsing et al., 2002), perhaps making the persistence of southern populations more vulnerable to changes. Understanding the consequences of landscape‐scale changes such as climate and wildfire on the distribution, density, and demography of snowshoe hares and their predators will continue to be important for wildlife conservation in northern North America.

## 5 Conclusions

Our gradient‐based (vs. type‐based) and multiscale approach embraced the current paradigm in ecology (e.g., Cushman et al., 2010; McGill, 2010) and advanced the understanding and management of snowshoe hare habitat. First, our analyses produced consistent patterns across scales and emphasized the importance of horizontal cover, spruce‐fir, and lodgepole pine as indicators of hare habitat within the mixed conifer context of the Northern Rockies. Second, we predicted snowshoe hare habitat and demonstrated that it is patchily distributed at a coarse scale within the Northern Rockies and that multiple‐use lands (e.g., national forests, state‐managed lands) are essential for the conservation of snowshoe hare habitat. Simply focusing on wilderness areas or national parks for conservation of snowshoe hares will likely result in ineffective strategies. Third, we provided explicit structural information concerning snowshoe hare habitat within the mixed conifer forests of the Northern Rockies that can be directly implemented by forest managers. The structure of forests with high use by snowshoe hares was characterized as dense (particularly in the understory), relatively closed, and multistoried, which we described using metrics such as quadratic mean diameter, trees/ha, canopy cover, and basal area (for additional metrics, see Table 2 and Table A1 in Appendix A). These stand characteristics can arise in nearly all successional stages and are presumably realized following disturbance agents (e.g., wildfire, insect damage, root disease, or cutting) of intermediate severity that allow patches of light to reach the forest floor. Overall, forest managers can apply this collective understanding to inform decision making relevant to habitat management of snowshoe hares and their associated predators within the Northern Rockies.

## Conflict of Interest

None declared.

## Figures and Tables

**Figure B1:**
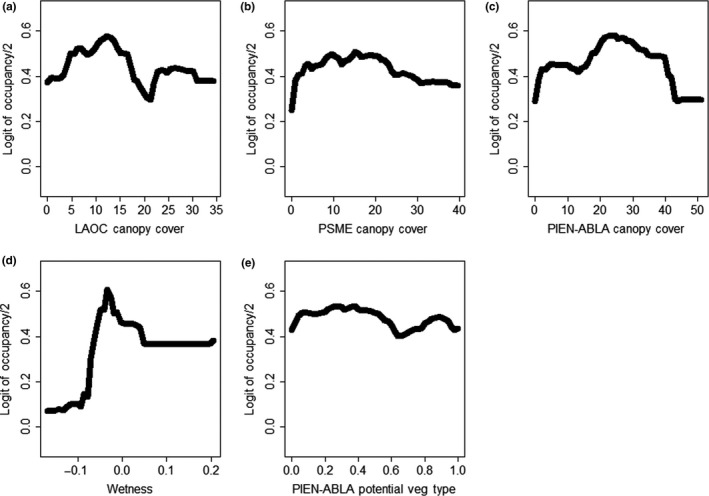
Partial dependence plots displaying the relationship between occupancy of snowshoe hares (*Lepus americanus*) and covariates (a–e) that were included in our top Random Forest model, but did not exhibit a consistent trend. See Table 1 for covariate descriptions

## Appendix A

### Results from quantile regression and summary of forest structural metrics

#### 1

**Table A1:** Mean (±95% CIs) tree density by size class (diameter at breast height; DBH) along the gradient of snowshoe hare (*Lepus americanus*), pellet density (pellet density = mean number of pellets/5.1 × 305 cm subplots), and the associated estimate of hare density (hares/ha) using the equation of Krebs, Boonstra et al. (2001). All metrics were calculated within the Forest Vegetation Simulator (FVS; Crookston & Dixon, 2005) from data collected using a 10 basal area factor prism, and only included trees ≥12.7 cm (i.e., 5 inches) in DBH. Both English and metric (italicized) units and values are provided; English and metric tree density are expressed as trees/acre and *trees/ha*, respectively

Pellet density	Hares/ha	Sample size	Tree density		
			DBH: 5–10 in,*12.7–25.4 cm*	DBH: 10*–*20 in,*25.4–50.8 cm*	DBH: 20*–*64 in,*50.8–162.56 cm*
None	0	432	121 (107*–*135)*299 (264–333)*	40 (36*–*44)*99 (88–109)*	3 (2*–*3)*6 (5–8)*
0.2–1.2	0.28	378	168 (152*–*184)*415 (375–455)*	51 (46*–*56)*126 (114–138)*	3 (2*–*3)*7 (5–8)*
1.2–2.4	0.81	158	193 (164*–*221)*476 (406–546)*	43 (36*–*50)*106 (90–123)*	3 (2*–*4) *7 (4–9)*
2.4–5.2	1.48	157	202 (173*–*231)*499 (428–570)*	37 (30*–*44)*91 (74–108)*	2 (1*–*2)*4 (2–6)*
5.2*–*40.4	4.21	172	197 (168*–*226)*487 (414–559)*	27 (20*–*34)*66 (50–83)*	1 (0.54*–*2) *3 (1–5)*
